# Identifying patterns of Long-COVID diagnosis pathways: a Latent Class Analysis of Welsh clinical data

**DOI:** 10.23889/ijpds.v9i5.2612

**Published:** 2024-09-18

**Authors:** Caroline Reid-Westoby, Jennifer Enns, Marni Brownell, Gilles Detillieux, Nathan Nickel, Magdalena Janus

**Affiliations:** 1 McMaster University; 2 University of Manitoba

Children with special health needs (SHN) may be more likely to experience mental disorders compared to their peers without SHN. The aim of this study was to establish and contextualize the risk of mental disorders among children flagged as having SHN at school entry. We linked data from the Early Development Instrument (EDI), a teacher-completed questionnaire of children’s developmental health in kindergarten, collected in Manitoba between 2006 and 2015, with provincial health administrative data up to 2019. Using binary logistic regressions, we examined the odds of receiving a diagnosis of any mental disorder, by categories of SHN: special needs, impairments in physical, vision/hearing, learning, speech, behaviour, and emotions, needing further assessment, and having 2+ SHN categories. Of the 36,462 children with EDI data linked to health administrative data, 5,882 (16.1%) were identified as having a SHN in kindergarten. The odds of developing a mental disorder varied by subtype of SHN. Children needing further assessment, with special needs, 2+ SHN categories, or with a learning, behavioural, or emotional impairment had between 1.35 and 3.27 times the odds of receiving a mental health diagnosis than their peers without these issues. Having a behavioural impairment increased a child’s odds the most. Having a physical, visual, or hearing impairment was not associated with a mental disorder. Having a special needs designation and impairments in behaviour and emotions in kindergarten puts children at risk of a future mental disorder. These findings may help generate wider mental health supports in schools for children with SHN.

